# Truncated Variants in *FAM20A* and *WDR72* Genes Underlie Autosomal Recessive Amelogenesis Imperfecta in Four Pakistani Families

**DOI:** 10.1007/s10528-025-11087-2

**Published:** 2025-03-19

**Authors:** Sadaqat Ullah, Sher Alam Khan, Samin Jan, Salah Ud Din, Nazif Muhammad, Zia Ur Rehman, Abid Jan, Muhammad Tariq, Noor Muhammad, Abdul Ghani, Naveed Wasif, Saadullah Khan

**Affiliations:** 1 Department of Biotechnology and Genetic Engineering, Kohat University of Science & Technology (KUST), Khyber Pakhtunkhwa, Kohat, Pakistan; 2 Department of Computer Science and Bioinformatics, Khushal Khan Khatak University, Karak, Pakistan; 3 Department of Medical Laboratory Technology, University College of Duba, University of Tabuk, Tabuk, Kingdom of Saudi Arabia; 4 Department of Chemistry, Kohat University of Science & Technology (KUST), Khyber Pakhtunkhwa, Kohat, Pakistan; 5 Institute of Human Genetics, Ulm University and Ulm University Medical Center, 89081 Ulm, Germany; 6 Institute of Human Genetics, University Hospital Schleswig-Holstein, Campus Kiel, Kiel, Germany

**Keywords:** Amelogenesis imperfecta, Consanguinity, Enamel defects, Exome sequencing, *FAM20A*, *WDR72*, Segregation analysis

## Abstract

Amelogenesis Imperfecta (AI) is a set of hereditary diseases affecting enamel development, leading to various types of enamel defects, potentially impacting oral health unassociated with other generalized defects. AI manifests in syndromic and non-syndromic forms and can be inherited through autosomal recessive, autosomal dominant, or X-linked inheritance patterns. Genetic studies have identified sequence variants in a number of genes (≥ 70) linked to both syndromic and non-syndromic AI, highlighting the genetic diversity underlying the condition. The current study involved clinical evaluation and exome sequencing, aimed at identifying the causative variants in four unrelated consanguineous Pakistani families presenting AI phenotypes. The exome sequencing results revealed a novel homozygous frameshift variant *FAM20A*: NM_017565.4, c.188dupA; p.(Asp63Glufs*17) in families A, B, and C while a nonsense homozygous variant *WDR72*: NM_182758.4, c.2686C > T; p. (Arg896*) in family D. The segregation of both variants was confirmed by Sanger sequencing. Bioinformatics analysis predicted the pathogenicity of these genetic variants. These alterations suggest functional consequences, potentially impairing the FAM20A and WDR72 proteins and causing dental anomalies. This investigation significantly broadens our understanding of *FAM20A* and *WDR72*’s involvement in AI. Furthermore, this study highlights the genetic heterogeneity of AI (involving *FAM20A* and *WDR72* in this study) within the Pakistani population.

**Supplementary Information:**

The online version contains supplementary material available at 10.1007/s10528-025-11087-2.

## Introduction

Amelogenesis Imperfecta (AI) is a hereditary condition, affecting the enamel of both primary and secondary teeth, leading to a range of dental conditions (DeSort 1983; Gadhia et al. 2012). Its prevalence is estimated to range from 1 in 700 to 1 in 14,000 individuals (Aldred et al. 2003; Chan et al. 2011; Mohan et al. 2021). It can exhibit sporadic, sex-linked, autosomal recessive, or dominant inheritance patterns and can exist in isolation or associated with other abnormalities (Guru et al. 2011; Eftekhar et al. 2022). AI is classified into various types based on gene or loci, dental abnormalities, and inheritance patterns (Supp. Table 1).

Sequence variants in the *FAM20A* (OMIM: 611062) have been associated with a unique autosomal recessive dental anomaly known as Amelogenesis Imperfecta-IG (AI1G; OMIM: 204690). This condition, also known as enamel-renal syndrome, is characterized by a distinct set of symptoms including nephrocalcinosis, gingival overgrowth, hypoplastic enamel on primary and secondary dentition, pulp stones, and delayed or unsuccessful secondary dentition eruption (Wang et al. 2013, 2014). Furthermore, variations in *WDR72* (OMIM: 613214) have been associated with type IIA3 (OMIM: 613211) also known as type II hypomaturation, characterized by hypoplastic pitted autosomal dominant/recessive enamel defects. (El-Sayed et al. 2009; Chamarthi et al. 2012). AI can occur in a proband in a family with a disease history from the maternal or paternal lineage (El-Sayed et al. 2009). It can also occur spontaneously in individuals not inherited from either of their parents (de novo mutation), i.e., having no family history of the disorder (Kim et al. 2021), and can affect both primary and secondary dentitions (McDonald et al. 2012).

The primary focus of this study was to identify the sequence variants responsible for the phenotypic conditions associated with AI in four genetically unrelated consanguineous Pakistani families. The genetic and molecular analysis revealed truncating variants in *FAM20A* and *WDR72*, thereby establishing a direct link between these mutations and the occurrence of AI.

## Materials and Methods

### Sample Collection and DNA Extraction

The current study encompasses clinical and molecular investigations of four genetically unrelated consanguineous Pakistani families harboring phenotypes associated with AI. The informed written consent of the parents were diligently obtained, and permission was granted for clinical and molecular assessments and the publication of relevant data and photographs. The guiding principles of the Declaration of Helsinki were followed.

This study was ethically approved by the “Research Ethical Committee” of Kohat University of Science & Technology, Kohat, Khyber Pakhtunkhwa (KP), Pakistan (dated September 5, 2023; Certificate number 1010).

**Fig. 1:**
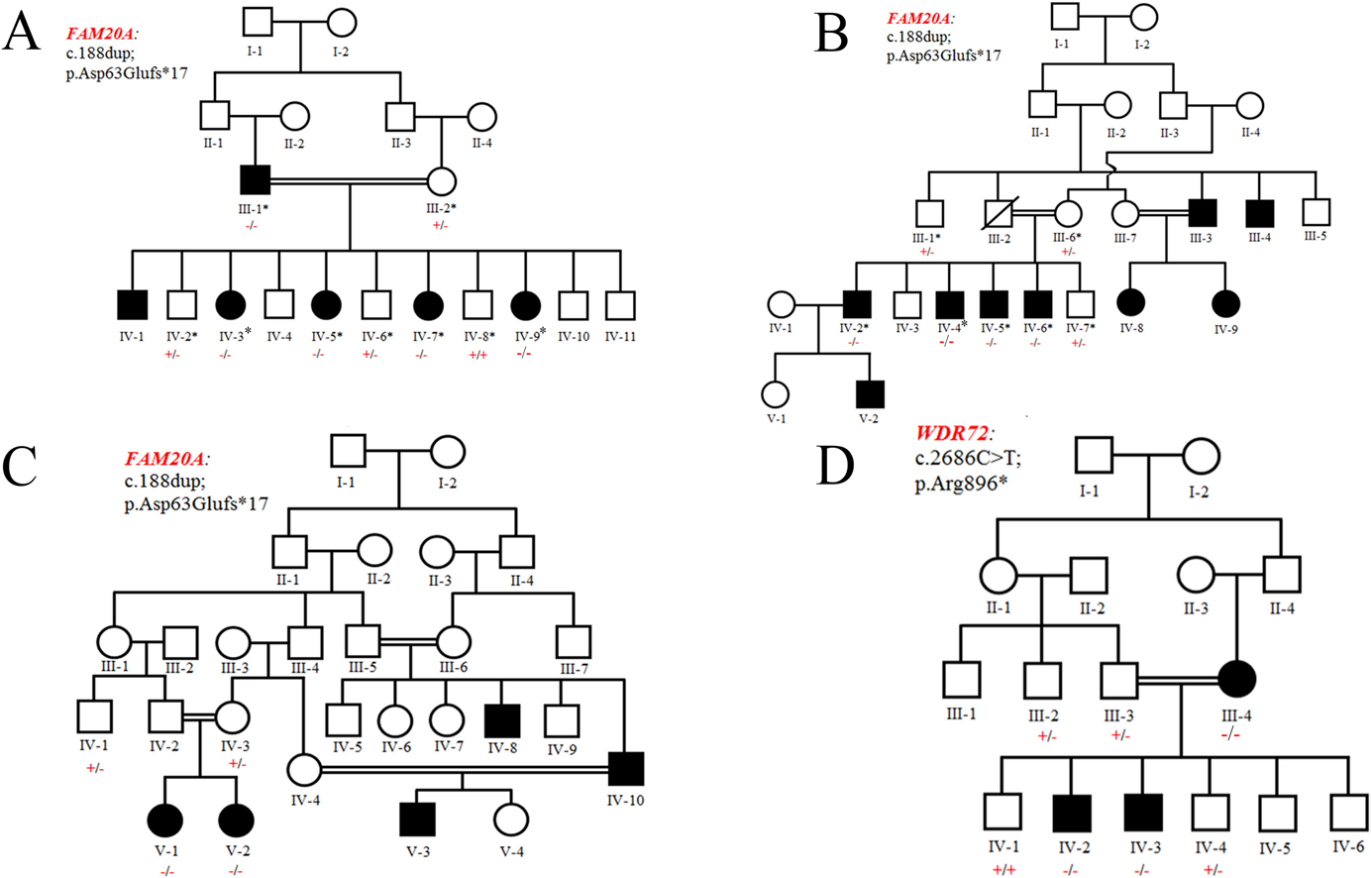
Pedigrees of families **A**–**D** demonstrating the autosomal recessive inheritance pattern of AI. Unfilled squares and circles represent unaffected female and male individuals, respectively. Filled symbols indicate affected individuals. Double parallel lines denote consanguinity. Asterisks mark the participants of the study. The symbol ‘+’ represents the wild-type allele, while ‘−’ indicates the mutant allele

The sample collection process was conducted, ensuring the inclusion of all individuals marked with asterisks in the pedigrees of the families (Fig. 1). Venous blood samples were collected in EDTA tubes. Genomic DNA was extracted from lymphocytes using the standard phenol chloroform method (Sanbrook et al. 1989), followed by DNA quantification using a Nanodrop spectrophotometer.

### Exome Sequencing and Data Analysis

One affected individual from each family, i.e., family A (III-1), family B (IV-2), family C (V-1) and (III-4) in family D were used for exome sequencing. Exome sequencing as previously described by Muhammad et al. (Muhammad et al. 2023), was conducted on a paired-end library using the Nextera DNA Exome kit (Illumina Inc., USA) following the manufacturer’s guidelines. Approximately 100 ng of genomic DNA was used for enzymatic fragmentation, followed by ligation of unique dual-indexed adapters and exome enrichment for an affected individual of each family. Sequencing was performed with a 75 × 2 paired-end setups using the NextSeq® 550/500 High Output Reagent Cartridge v2 kit on the Illumina NextSeq500 platform (Illumina, San Diego, CA, USA).

The initial data were processed using Illumina’s real-time analysis (RTA) software v1.8. Sequence reads were aligned to the human reference genome build GRCh37/hg19 (http://www.genome.ucsc.edu/) using the BWA-SW alignment tool (Li et al. 2010). Samtools v0.1.9 (Li et al. 2009) was used to convert the sequence alignment map (SAM) files into binary alignment map (BAM) files. Picard tools were employed to enhance read quality, while the Genome Analysis Toolkit (GATK) handled realignment and recalibration of base quality scores. Single nucleotide polymorphisms (SNPs) and short insertions/deletions (INDELs) were identified using Platypus (Rimmer et al. 2014), HaplotypeCaller (McKenna et al. 2010), and Mpileup programs. Further variant filtration was performed through GATK’s variant quality score recalibration (VQSR). CNV detection was performed using the CNMOPS and ExomeDepth algorithms (Klambauer et al. 2012). The COMBINE and FUNC algorithms (varpipe_v2.26, https://varbank.ccg.uni-koeln.de/) were used to integrate and annotate functional variants in the data.

Exome sequencing data were analyzed using the Varvis exome analysis pipeline (Limbus Medical Technologies GmbH), achieving a mean coverage of more than 92% at 20X and 96.6% at 10X for the targeted regions. Variants were filtered to identify homozygous or compound heterozygous mutations in genes associated with autosomal recessive AI as listed in the OMIM database. All homozygous and potential compound heterozygous variants in known autosomal recessive AI genes with an allele frequency of < 1%, while heterozygous variants with an allele frequency of ≤ 0.1% were examined from the AI gene panel (Supp. Table 3). Additionally, a minor allele frequency (MAF) cutoff of less than 0.5% was applied using the gnomAD database.

### Segregation Analysis

Suspected disease variants in *FAM20A* and *WDR72* were submitted for Sanger sequencing using the available DNA samples to validate their co-segregation with a disease phenotype in all four families (A-D). To locate the exact variation site in the genes, the Ensembl genome browser (www.ensembl.org) was used to retrieve genomic regions, including exons, introns, and upstream and downstream untranslated regions (UTRs). The online Primer3 tool (https://primer3.ut.ee/) was used to design the following primers.

*FAM20A*: Forward primer: 5′-GCTTCGGATCGGGAGTT-3′ and Reverse primer: 5′-TTCTTGCGCCTTTTCTCC-3′; *WDR72*: Forward primer: 5′-TGTTGCCAGGTTGGGATT-3′ and Reverse primer: 5′-CCCCACTGGAAAGAAGGAA-3′.

### In silico Analysis

Pathogenicity and functional implications of genetic variants were predicted using four bioinformatic tools: MutationTaster (https://www.mutationtaster.org/), VarSome (https://varsome.com/), CADD (https://cadd.gs.washington.edu/snv), and InterVar (https://wintervar.wglab.org/).

## Results

### Clinical Phenotypes and Pedigrees

Four ethnically matched families, A, B, C, and D were recruited from remote regions of Kohat district, Khyber Pakhtunkhwa (KP), Pakistan. All the patients were clinically diagnosed, characterized and managed by the dentist in the district headquarters hospital (DHQ) Kohat, Khyber Pakhtunkhwa, Pakistan. The clinical features of the affected individuals are also explained in Supp. Table 2.

### Family A

**Fig. 2:**
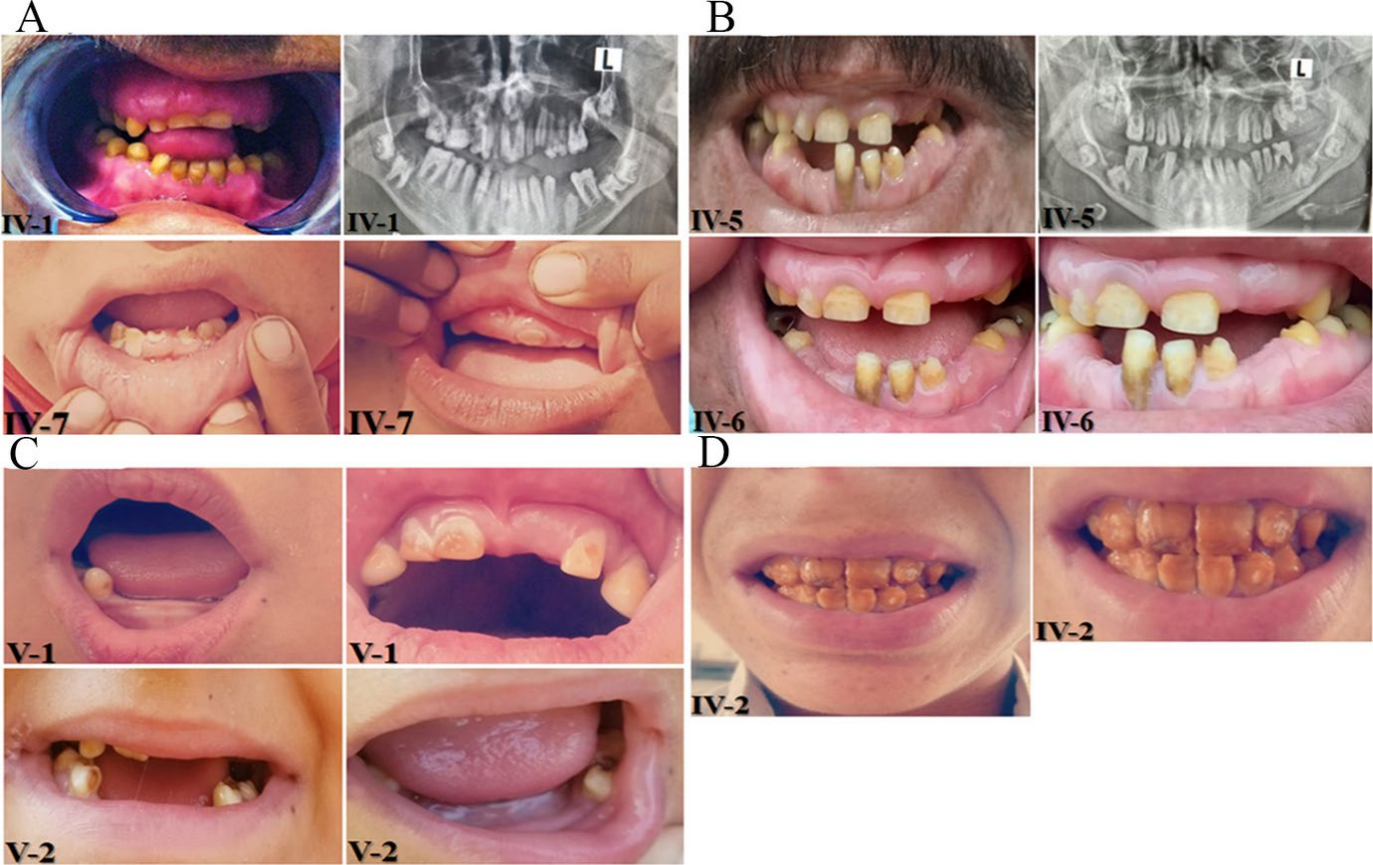
Phenotypic characteristics of the affected individuals in each family. Family A (**A**): Shows the phenotypic characteristics of the affected individuals of family A. IV-7 is a 6-year-old affected girl showing small, yellow, pointed, spaced teeth with small or absent enamel. IV-5 is a 16-year-old girl with irregular, small, yellow, spotted teeth; the upper enamel is almost absent. Family B (**B**): shows the phenotypic characteristics of the affected individuals of family B. IV-5 is a 15-year-old affected male with yellow, small, or absent enamel. IV-5 and IV-4 are affected male individuals, aged 18 and 23, respectively, exhibiting small, spaced, spotted enamel. IV-2 is a 28-year-old affected male with irregular, thin, yellow, hypoplastic, or absent enamel. Family C (**C**): shows the phenotypic characteristics of the affected individuals of family C. V-1 is a 6-year-old affected girl showing small, yellow, grooved first molars and premolars, with absent central and lateral incisors, as well as canines (cuspid). Family D (**D**): phenotypic characteristics of the affected individuals of family D. IV-2 is an affected male of years showing defective enamel with normal thickness, severely hypomineralized, susceptible to attrition, and pitted or grooved

Family A was a consanguineous family spanning four generations having six affected individuals (III-1, IV-1, IV-3, IV-5, IV-7, and IV-9) (Fig. 1A), but only five of them (III-1, IV-3, IV-5, IV-7, and IV-9) were available for blood collection. The family had no prior history of any other genetic abnormality. Affected members showed variable dental features recorded as small, thin, pitted, grooved, or rough spaced pointed yellowish-brown enamel, hypomineralized (insufficient mineral content) or hypoplastic (inadequately developed), hypodontia, microdontia, absent or thin hypoplastic enamel, abnormal cusps, and delayed eruption (Fig. 2A).

### Family B

The Family B pedigree showed a consanguineous family spanning five generations with nine affected individuals (III-3, III-4, IV-2, IV-4, IV-5, IV-6, IV-8, IV-9, V2) (Fig. 1B), but only four of them (IV-2, IV-4, IV-5, and IV-6) took part in the study. Affected individuals showed discolored upper and lower teeth, gingival recession, gingival hyperplasia, plaque accumulation, sensitivity in posterior teeth, attrition, loss of clinical crown height along with pits (Fig. 2B).

### Family C

Family C also showed a five-generation consanguineous pedigree with five affected individuals (IV-8, IV-10, V-1, V-2, and V-3) but only two (V-1, V-2) of them gave consent to participate in this study (Fig. 1C). Hypodontia, microdontia, yellow–brown mottles, or spotted teeth clinical phenotypes were observed in these affected members (Fig. 2C).

A detailed clinical examination of affected individuals from the three families (A, B, and C) showed no indication of any associated abnormalities, including renal malfunctioning. Hematological tests (serum creatinine, blood urea nitrogen, estimated GFR, serum potassium, serum sodium, serum calcium, and phosphorus) and urine analysis (urinalysis, urine protein-to-creatinine ratio, urine specific gravity and urine pH) were undertaken in accordance with the reference values to investigate the nephrological irregularities but showed no indication of any associated abnormalities, including renal dysfunction.

### Family D

The family D showed a four-generation consanguineous pedigree with three affected individuals (III-4, IV-2, IV-3) (Fig. 1D), showing orange-brown teeth as a major clinical feature with standard enamel thickness, hypomineralized teeth susceptible to attrition, pitted, and grooved (Fig. 2D).

### Genetic Screening

**Fig. 3:**
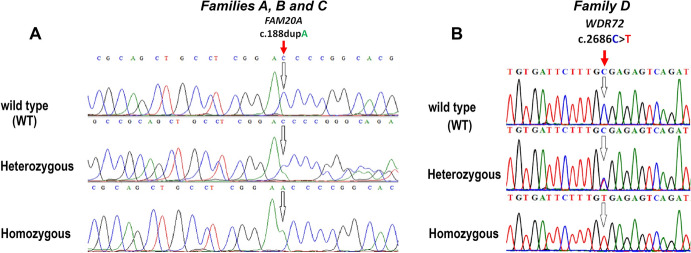
Chromatograms of partial DNA sequences showing identified variants in the *FAM20A* and *WDR72* genes. **A** The chromatogram for a partial DNA sequence corresponding to the *FAM20A* gene reveals the presence of a homozygous frameshift variant, c.188dup; p.(Asp63Glufs*17), in families A, B, and C. The upper panel displays a homozygous wild-type individual, the middle panel shows a heterozygous carrier, and the lower panel displays a homozygous affected individual. The arrow indicates the mutation point. **B** The chromatogram for a partial DNA sequence corresponding to the *WDR72* gene shows the presence of a homozygous nonsense variant, c.2686C > T; p.(Arg896*), in family D. The upper panel displays a homozygous wild-type individual, the middle panel shows a heterozygous carrier, and the lower panel shows a homozygous affected individual. The arrow indicates the mutation point

Using exome sequencing followed by the stringent filtering processes, we identified a frameshift variant in the *FAM20A* and a homozygous nonsense variant in the *WDR72*. An in-house AI gene panel (supp. Table 3), covering autosomal dominant, autosomal recessive, and X-linked genes involved in both syndromic and non-syndromic forms of AI, did not reveal any rare variants other than a homozygous frameshift variant, *FAM20A*: NM_017565.4, c.188dupA; p.(Asp63Glufs*17) in exon-1 in the families A, B, and C while a nonsense homozygous variant *WDR72*: NM_182758.4, c.2686C > T; p.(Arg896*) with its chromosome coordinate (GRCh38/hg38) chr15:53907717G > A in exon 15 in family D. Moreover, Sanger sequencing confirmed the segregation of the *FAM20A* variant c.188dup; p.(Asp63Glufs*17) with its chromosome coordinate (GRCh38/hg38) chr17:66596619G > GT in families A, B, and C. All the affected individuals were homozygous but their parents were heterozygous (Fig. 3A). *WDR72* variant c.2686C > T; p.(Arg896*) was segregating in family D with the disease phenotypes in an autosomal recessive manner. All the affected individuals were homozygous (^T^/^T^) but their parents were heterozygous (^C^/^T^) for the *WDR72* variant (Fig. 3B).

The identified variant c.188dupA; p.(Asp63Glufs*17) in *FAM20A* has not been reported in control databases like gnomAD (https://gnomad.broadinstitute.org/), HGMD 2024 (https://www.hgmd.cf.ac.uk/) and PGMD 2024 (https://pakmutation.mikesoft.com.pk/) (Qasim et al. 2018), while *WDR72* gene variant c.2686C > T; p. (Arg896*) has been reported pathogenic in all these databases (El-Sayed et al. 2011).

### In silico analysis

*In-silico* tools, including MutationTaster, and VarSome were utilized to analyze the functional impacts of the novel frameshift variant (*FAM20A*: c.188dupA) in families A, B, and C and the nonsense variant (*WDR72*: c.2686C > T) in family D. InterVar (https://wintervar.wglab.org/) was used to classify both the *FAM20A* and *WDR72* variants based on the ACMG/AMP 2015 guidelines, classifying them as pathogenic (PVS1, PM2, PP3, PP1, PP4, and PP5) (Li et al. 2017) (Supp. Table 4).

## Discussion

AI is a group of rare hereditary anomalies that affect enamel formation, leading to various dental disease conditions (Witkop 1988). In this study, we investigated four Pakistani autosomal recessive AI families, using whole exome sequencing. Upon analysis, truncating variants were identified in *FAM20A* and *WDR72*.

*FAM20A* is mapped to chromosome 17q24.2, and contains 11 exons. It encodes a 541 amino acids long FAM20A protein (family with sequence similarity 20, member A) (Cherkaoui Jaouad et al. 2015). FAM20A has high expression in gingiva and ameloblasts and plays a fundamental role in enamel development and gingival homeostasis (O’Sullivan et al. 2011). A highly conserved insertion in the Gly-rich loop is residing in FAM20A, that plays a critical role in maintaining the function of FAM20A (Xiao et al. 2013). The FAM20 family consists of three kinases: *FAM20A*, *FAM20B* (OMIM: 611,063; 1q25.2), and *FAM20C* (OMIM: 611,061; 7p22.3). The Kinase core of FAM20A is structurally similar to that of ceFam20 (Caenorhabditis elegans ortholog of FAM20C). FAM20A displays an unusual disulfide pattern created by a pair of cysteine residues within the conserved insertion region in the Gly-rich loop. FAM20A is a pseudokinase in the secretory pathway and facilitates a deeper understanding of AI caused by *FAM20A* variants (Cui et al. 2017). Truncation of this conserved region results in aberrant mRNA, leading to AI phenotypes (Cho et al. 2012). These three proteins are highly conserved in humans, rats, and mice, and their significant expression levels have been observed during hematopoiesis (Nalbant et al. 2005). FAM20A, or family with sequence similarity 20 member A, regulates mineralization in various tissues, particularly in the process of amelogenesis, the formation of the enamel covering a tooth’s crown (Nalbant et al. 2005; Martelli-Júnior et al. 2008; O’Sullivan et al. 2011).

In this study, a novel *FAM20A* frameshift variant (c.188dup) was identified in three families (A, B and C), leading to a premature stop codon p.(Asp63Glufs*17) in the mRNA transcript. The nonsense-mediated mRNA decay (NMD) pathway will be activated and this premature truncated mRNA will be degraded, which likely disrupts FAM20A’s role in enamel formation and biomineralization. The *FAM20A* frameshift variant segregates in three families (A, B, and C), which suggests a possible founder effect, because all the three families belong to the same population of district Kohat from the southern region of Khyber Pakhtunkhwa, Pakistan.

The clinical manifestations observed in individuals carrying this mutation are consistent with those previously reported for *FAM20A*-related AI (O’Sullivan et al. 2011; Cabral et al. 2013; Cherkaoui Jaouad et al. 2015). Affected members of these three families showed a range of enamel defects, such as thin, pitted, grooved, or discolored enamel, and hypomineralized and hypoplastic enamel. Other issues included hypodontia, microdontia, abnormal cusps, and delayed tooth eruption. These features are consistent with AI caused by *FAM20A* mutations.

Furthermore, a comprehensive clinical evaluation of all the patients in families A, B, and C showed no evidence of renal abnormalities.

*WDR72* (tryptophan-aspartate repeat domain 72) is located on 15q21.3, spanning approximately 250 kb and consisting of 20 exons. *WDR72* encodes 1102 amino acids lengthy protein called WD repeat containing protein 72 (Katsura et al. 2014). WDR72 is a member of the WD40-repeat domain super family. Proteins in the WD40-repeat domain family have 4–8 repeating units of about 44–60 residues ending in tryptophan and aspartic acid in their N-termini. WD40 proteins contain several repeat domains that encode a series of anti-parallel β-sheet blades, called a “β-propeller” (Stirnimann et al. 2010; Xu et al. 2011). Proteins containing these β-propellers are observed in a broad range of cell processes, including signal transduction, cell cycle regulation, and vesicular trafficking (Good et al. 2011). WDR72 has 8 WD40-repeat domains (WD1 to WD8) (https://www.uniprot.org/uniprotkb/Q3MJ13/entry#family_and_domains). As El-Sayed et al. predicted, the protein structure indicates characteristics typical proteins involved in membrane deformation (El-Sayed et al. 2009). Its significance in enamel mineralization is highlighted, potentially through its involvement in endocytic vesicle trafficking, as suggested by Katsura et al. (2014) and El-Sayed et al. (2009) (El-Sayed et al. 2009; Katsura et al. 2014).

WDR72 protein linked to synaptic vesicle mobility and Ca^2+^-dependent exocytosis, share similarities. *WDR72* is broadly expressed across various tissues, including detection in the enamel organ of growing mouse incisors, where maturation ameloblasts exhibit more pronounced staining compared to secretory ameloblasts (El-Sayed et al. 2009). Furthermore, *WDR72* expression was also found in some connective tissue and bone cells (El-Sayed et al. 2009).

Sequence variants in the *WDR72* have been associated with certain dental conditions, including AI, type IIA3, characterized by defects in enamel formation, leading to a range of dental abnormalities such as discoloration, pitting and increased vulnerability to tooth decay (El-Sayed et al. 2009; Lee et al. 2010; Katsura et al. 2014). In this case, we identified a recurrent nonsense variant in family D in the *WDR72*: c.2686C > T previously reported by El-Sayed et al. and Rungroj et al. (El-Sayed et al. 2011; Rungroj et al. 2018), resulting in the truncated protein p. (Arg896*). It was a novel homozygous nonsense variant identified by El-Sayed et al. in a Pakistani family, but in the case of Rungroj et al. it was a recurrent variant identified in consanguineous Indian family. The *WDR72* variant c.2686C > T (p.Arg896*) occurs within the β-propeller domain, a crucial region for the protein’s function. This variant introduces a premature stop codon, which is likely to trigger NMD, leading to the degradation of the mRNA. This is consistent with the pathogenic mechanism seen in case of other nonsense variants in *WDR72*, contributing to the onset of AI. The affected family members carrying the c.2686C > T mutation displayed distinct dental phenotypes, including orange-brown teeth with significantly hypomineralized enamel prone to attrition, alongside pitted and grooved surfaces. These clinical features are consistent with *WDR72*-related AI, reflecting its crucial role in enamel mineralization and integrity, with the identified mutation contributing to loss of function through protein truncation.

This is a machine and software-based study and lacks functional analysis. For more information and research on the identified gene variants, functional studies in model organisms will be essential.

## Conclusions

This study identifies a novel homozygous frameshift variant, *FAM20A*: c.188dup; p.(Asp63Glufs*17), in families A, B, and C, and a homozygous nonsense variant, *WDR72*: c.2686C > T p.(Arg896*), in family D, within four AI families from the Pashtun ethnicity in Khyber Pakhtunkhwa, Pakistan. These findings highlight the crucial roles of *FAM20A* and *WDR72* in dental development. While functional studies are lacking, we anticipate that future research will provide deeper insights into these variants and tooth morphogenesis.

## Supplementary Information

Below is the link to the electronic supplementary material.

Supplementary file1 (DOCX 111 KB)—Supplementary Table 1. The classification of the amelogenesis imperfecta into various types based on gene or loci

Supplementary file2 (DOCX 19 KB)—Supplementary Table 2. Phenotypic characteristics of the affected individuals in families A, B, C, and D

Supplementary file3 (DOCX 19 KB)—Supplementary Table 3. In-house AI gene panel used to detect the causative disease variants in AI patient during exome sequencing

Supplementary file4 (DOCX 14 KB)—Supplementary Table 4. Pathogenicity prediction of FAM20A and WDR72 variants by using different tools

## Data Availability

The raw data (sequence, photographs, and pedigrees) is stored in the password-protected computer at Kohat University of Science and Technology, Kohat, and is available upon request. We have submitted the identified variants data to ClinVar (https://submit.ncbi.nlm.nih.gov/clinvar/) under the following accession numbers (SCV005061849, SCV005061850).
