# Supplementary material for: Truncated Variants in FAM20A and WDR72 Genes Underlie Autosomal Recessive Amelogenesis Imperfecta in Four Pakistani Families
Source: Biochem Genet. 2025 Mar 19;64(1):1311–23. doi: 10.1007/s10528-025-11087-2 (PMC12882964; doi:10.1007/s10528-025-11087-2)
Supplement: Supplementary file 1 — Supplementary file1 (DOCX 111 KB)—Supplementary Table 1. The classification of the amelogenesis imperfecta into various types based on gene or loci [file 10528_2025_11087_MOESM1_ESM.docx]

**Supplementary table 1.** The classification of the amelogenesis imperfecta into various types based on gene or loci.

| **S. No.** | **Gene** | **OMIM** | **Cytogenic location** | **Type of AI** | **OMIM** | **Non-syndromic/Syndromic** | **Inheritance** | **Reference** |
| --- | --- | --- | --- | --- | --- | --- | --- | --- |
| **1.** | *AMELX* | 300391 | Xp22.2 | AI1E | 301200 | Non-syndromic | XLD | Lagerström et al. (1991) |
| **2.** | *ENAM* | 606585 | 4q13.3 | AI1B | 104500 | Non-syndromic | AD | Rajpar et al. (2001) |
|  |  |  |  | AI1C | 204650 | Non-syndromic | AR | Hart et al. (2003) |
| **3.** | *KLK4* | 603767 | 19q13.41 | AI2A1 | 204700 | Non-syndromic | AR | Hart et al. (2004) |
| **4.** | *DLX3* | 600525 | 17q21.33 | AI4 | 104510 | Non-syndromic/ syndromic | AD | (Dong et al. 2005; Bonnet et al. 2020) |
| **5.** | *MMP20* | 604629 | 11q22.2 | AI2A2 | 612529 | Non-syndromic | AR | Kim et al. (2005) |
| **6.** | *FAM83H* | 611927 | 8q24.3 | AI3A | 130900 | Non-syndromic | AD | Kim et al. (2008) |
| **7.** | *WDR72* | 613214 | 15q21.3 | AI2A3 | 613211 | Syndromic | AR | El-Sayed et al. (2009) |
| **8.** | *FAM20A* | 611062 | 17q24.2 | AI1G | 204690 | Syndromic/Non-syndromic | AR | (O'Sullivan et al. 2011; Jaureguiberry et al. 2012; Wang et al. 2013) |
| **9.** | *ODAPH/* C4orf26 | 614829 | 4q21.1 | AI2A4 | 614832 | Non-syndromic | AR | Parry et al. (2012) |
| **10.** | *SLC24A4* | 609840 | 14q32.12 | AI2A5 | 615887 |  | AR | Parry et al. (2013) |
| **11.** | *LAMB3* | 150310 | 1q32.2 | AI1A | 104530 | Non-syndromic/ syndromic | AD | (Kim et al. 2013; Poulter et al. 2014b; Lee et al. 2015) |
| **12.** | *ITGB6* | 147558 | 2q24.2 | AI1H | 616221 | Non-syndromic | AR | (Poulter et al. 2014a; Wang et al. 2014) |
| **13.** | *ACPT /ACP4* | 606362 | 19q13.33 | AI1J | 617297 |  | AR | Seymen et al. (2016) |
| **14.** | *GPR68* | 601404 | 14q32.11 | AI2A6 | 617217 |  | AR | Parry et al. (2016) |
| **15.** | *AMTN* | 610912 | 4q13.3 | AI3B | 617607 |  | AD | Smith et al. (2016) |
| **16.** | *RELT* | 611211 | 11q13.4 | AI3C | 618386 |  | AR | Kim et al. (2019) |
| **17.** | *SP6* | 608613 | 17q21.32 | AI1K | 620104 |  | AD | Smith et al. (2020) |
| **18.** | *AMBN* | 601259 | 4q13.3 | AI1F | 616270 |  | AR | (Poulter et al. 2014c; Prasad et al. 2016) |
| **19.** | *LAMA3* | 600805 | 18q11.2 | Hypoplastic pitted AI | ? |  | AD | (Bloch-Zupan et al. 2023) |
| **20.** | *LAMC2* | 150292 | 1q25.3 | Hypoplastic pitted AI | ? |  | AD |  |
| **21.** | *COL17A1* | 113811 | 10q25.1 | Hypoplastic pitted AI | ? | Syndromic | AR | (McGrath et al. 1996; Bloch-Zupan *et al.* 2023; Kim et al. 2023) |
|  | *AIRE* | 607358 | 21q22.3 | Hypoplastic AI with immune deficit | ? |  | AR | (Bloch-Zupan *et al.* 2023) |
| **22.** | *STIM1* | 605921 | 11p15.4 | Hypoplastic AI with clinical signs within the head, neck region, skin, nail, hair defects and immune deficit | ? |  | AD | (Feske et al. 2010) |
| **23.** | *ORAI1* | 610277 | 12q24.31 |  |  |  | AR |  |
| **24.** | CLDN19 | 610036 | 1p34.2 | Hypoplastic AI with genitourinary defects | ? |  | AR | Yamaguti et al. (2017) |
| **25.** | CLDN16 | 603959 | 3q28 |  |  |  | AR | (Bloch-Zupan *et al.* 2023) |
| **26.** | TSC1 | 605284 | 9q34.13 |  |  |  | AD |  |
| **27.** | TSC2 | 191092 | 16p13.3 |  |  |  | AD |  |
| **29.** | LTBP3 | 602090 | 11q13.1 | Hypoplastic AI, short stature, cardiovascular diseases | 601216 |  | AR | (Huckert et al. 2015; Nawaz et al. 2024) |
| **30.** | *TGFBR2* | 190182 | 3p24.1 | Hypoplastic AI with cardiovascular diseases | ? |  | AD | (Bloch-Zupan *et al.* 2023) |
| **31.** | *GALNS* | 612222 | 16q24.3 | Hypoplastic AI, pits, with skeletal defects | ? |  | AR |  |
| **32.** | *TSC1* | 605284 | 9q34.13 | Hypoplastic AI with clinical signs within the head, neck region, skin, nail and hair defects | ? |  | AD |  |
| **33.** | *TSC2* | 191092 | 16p13.3 |  | ? |  | AD |  |
| **34.** | *TP63* | 603273 | 3q28 |  | ? |  | AD |  |
| **35.** | *MSX2* | 123101 | 5q35.2 | Hypoplastic AI with clinical signs within the head, neck region, genitourinary defects | ? | Syndromic | AD |  |
| **36.** | *FAM20C* | 611061 | 7p22.3 |  | ? |  | AR |  |
|  | *RAI1* | 607642 | 17p11.2 |  | ? |  | AD |  |
| **37.** | *ARHGAP6* | 300118 | Xp22.2 | Hypoplastic AI with clinical signs within the head, neck region, skin, nail, hair defects and with neurological issues | ? |  | AD |  |
| **38.** | *PEX1* | 602136 | 7q21.2 |  | ? |  | AR |  |
| **39.** | *PEX2* | 170993 | 8q21.13 |  | ? |  | AR |  |
| **40.** | *PEX26* | 608666 | 22q11.21 |  | ? |  |  |  |
|  |  |  |  |  | ? | Syndromic | AR | (Bloch-Zupan *et al.* 2023) |
| **41.** | *SLC13A5* | 608305 | 17p13.1 | Hypoplastic AI with neurological issues | ? |  | AR |  |
| **42.** | *ATP6V1A* | 607027 | 3q13.31 | Hypoplastic AI with clinical signs within the head, neck region, skin, nail, hair defects and neurological issues | ? |  | AR |  |
| **43.** | *TGFBR2* | 190182 | 3p24.1 | Hypoplastic, Loeys-Dietz syndrome | ? |  | AD | (Mátyás et al. 2006) |
| **44** | *PORCN* | 300651 | Xp11.23 | Hypoplastic AI with skin, nail, hair defects | ? |  | XLD | (Bloch-Zupan *et al.* 2023) |
| **45.** | *COL7A1* | 120120 | 3p21.31 | Hypoplastic pitted AI | ? | Non-syndromic | AD | (Bloch-Zupan *et al.* 2023) |
| **46.** | *ROGDI* | 614574 | 16p13.3 | Hypomature AI, Kohlschutter Tonz syndrome | ? | Syndromic | AR | (Huckert et al. 2014; Bloch-Zupan *et al.* 2023) |

**Abbreviations:** XLD: X-linked dominant, AD: autosomal dominant, AR: autosomal recessive, AI: amelogenesis imperfecta

Bloch-Zupan, A., Rey, T., Jimenez-Armijo, A., Kawczynski, M., Kharouf, N., Dure-Molla, M., Noirrit, E., Hernandez, M., Joseph-Beaudin, C., Lopez, S., Tardieu, C., Thivichon-Prince, B., Dostalova, T., Macek, M., Jr., Alloussi, M. E., Qebibo, L., Morkmued, S., Pungchanchaikul, P., Orellana, B. U., Manière, M. C., Gérard, B., Bugueno, I. M., and Laugel-Haushalter, V. (2023). Amelogenesis imperfecta: Next-generation sequencing sheds light on Witkop's classification. *Frontiers in Physiology, 14*, 1130175. doi:10.3389/fphys.2023.1130175

Bonnet, A. L., Sceosole, K., Vanderzwalm, A., Silve, C., Collignon, A. M., and Gaucher, C. (2020). "Isolated" Amelogenesis Imperfecta Associated with DLX3 Mutation: A Clinical Case. *Case Rep Genet, 2020*, 8217919. doi:10.1155/2020/8217919

Dong, J., Amor, D., Aldred, M. J., Gu, T., Escamilla, M., and MacDougall, M. (2005). DLX3 mutation associated with autosomal dominant amelogenesis imperfecta with taurodontism. *American Journal of Medical Genetics. Part A, 133a*(2), 138-141. doi:10.1002/ajmg.a.30521

El-Sayed, W., Parry, D. A., Shore, R. C., Ahmed, M., Jafri, H., Rashid, Y., Al-Bahlani, S., Al Harasi, S., Kirkham, J., Inglehearn, C. F., and Mighell, A. J. (2009). Mutations in the beta propeller WDR72 cause autosomal-recessive hypomaturation amelogenesis imperfecta. *American Journal of Human Genetics, 85*(5), 699-705. doi:10.1016/j.ajhg.2009.09.014

Feske, S., Picard, C., and Fischer, A. (2010). Immunodeficiency due to mutations in ORAI1 and STIM1. *Clinical Immunology, 135*(2), 169-182. doi:10.1016/j.clim.2010.01.011

Hart, P. S., Hart, T. C., Michalec, M. D., Ryu, O. H., Simmons, D., Hong, S., and Wright, J. T. (2004). Mutation in kallikrein 4 causes autosomal recessive hypomaturation amelogenesis imperfecta. *Journal of Medical Genetics, 41*(7), 545-549. doi:10.1136/jmg.2003.017657

Hart, T. C., Hart, P. S., Gorry, M. C., Michalec, M. D., Ryu, O. H., Uygur, C., Ozdemir, D., Firatli, S., Aren, G., and Firatli, E. (2003). Novel ENAM mutation responsible for autosomal recessive amelogenesis imperfecta and localised enamel defects. *Journal of Medical Genetics, 40*(12), 900-906. doi:10.1136/jmg.40.12.900

Huckert, M., Mecili, H., Laugel-Haushalter, V., Stoetzel, C., Muller, J., Flori, E., Laugel, V., Manière, M. C., Dollfus, H., and Bloch-Zupan, A. (2014). A Novel Mutation in the ROGDI Gene in a Patient with Kohlschütter-Tönz Syndrome. *Molecular Syndromology, 5*(6), 293-298. doi:10.1159/000366252

Huckert, M., Stoetzel, C., Morkmued, S., Laugel-Haushalter, V., Geoffroy, V., Muller, J., Clauss, F., Prasad, M. K., Obry, F., Raymond, J. L., Switala, M., Alembik, Y., Soskin, S., Mathieu, E., Hemmerlé, J., Weickert, J. L., Dabovic, B. B., Rifkin, D. B., Dheedene, A., Boudin, E., Caluseriu, O., Cholette, M. C., McLeod, R., Antequera, R., Gellé, M. P., Coeuriot, J. L., Jacquelin, L. F., Bailleul-Forestier, I., Manière, M. C., Van Hul, W., Bertola, D., Dollé, P., Verloes, A., Mortier, G., Dollfus, H., and Bloch-Zupan, A. (2015). Mutations in the latent TGF-beta binding protein 3 (LTBP3) gene cause brachyolmia with amelogenesis imperfecta. *Human Molecular Genetics, 24*(11), 3038-3049. doi:10.1093/hmg/ddv053

Jaureguiberry, G., De la Dure-Molla, M., Parry, D., Quentric, M., Himmerkus, N., Koike, T., Poulter, J., Klootwijk, E., Robinette, S. L., Howie, A. J., Patel, V., Figueres, M. L., Stanescu, H. C., Issler, N., Nicholson, J. K., Bockenhauer, D., Laing, C., Walsh, S. B., McCredie, D. A., Povey, S., Asselin, A., Picard, A., Coulomb, A., Medlar, A. J., Bailleul-Forestier, I., Verloes, A., Le Caignec, C., Roussey, G., Guiol, J., Isidor, B., Logan, C., Shore, R., Johnson, C., Inglehearn, C., Al-Bahlani, S., Schmittbuhl, M., Clauss, F., Huckert, M., Laugel, V., Ginglinger, E., Pajarola, S., Spartà, G., Bartholdi, D., Rauch, A., Addor, M. C., Yamaguti, P. M., Safatle, H. P., Acevedo, A. C., Martelli-Júnior, H., dos Santos Netos, P. E., Coletta, R. D., Gruessel, S., Sandmann, C., Ruehmann, D., Langman, C. B., Scheinman, S. J., Ozdemir-Ozenen, D., Hart, T. C., Hart, P. S., Neugebauer, U., Schlatter, E., Houillier, P., Gahl, W. A., Vikkula, M., Bloch-Zupan, A., Bleich, M., Kitagawa, H., Unwin, R. J., Mighell, A., Berdal, A., and Kleta, R. (2012). Nephrocalcinosis (enamel renal syndrome) caused by autosomal recessive FAM20A mutations. *Nephron. Physiology, 122*(1-2), 1-6. doi:10.1159/000349989

Kim, J. W., Lee, S. K., Lee, Z. H., Park, J. C., Lee, K. E., Lee, M. H., Park, J. T., Seo, B. M., Hu, J. C., and Simmer, J. P. (2008). FAM83H mutations in families with autosomal-dominant hypocalcified amelogenesis imperfecta. *American Journal of Human Genetics, 82*(2), 489-494. doi:10.1016/j.ajhg.2007.09.020

Kim, J. W., Seymen, F., Lee, K. E., Ko, J., Yildirim, M., Tuna, E. B., Gencay, K., Shin, T. J., Kyun, H. K., Simmer, J. P., and Hu, J. C. (2013). LAMB3 mutations causing autosomal-dominant amelogenesis imperfecta. *Journal of Dental Research, 92*(10), 899-904. doi:10.1177/0022034513502054

Kim, J. W., Simmer, J. P., Hart, T. C., Hart, P. S., Ramaswami, M. D., Bartlett, J. D., and Hu, J. C. (2005). MMP-20 mutation in autosomal recessive pigmented hypomaturation amelogenesis imperfecta. *Journal of Medical Genetics, 42*(3), 271-275. doi:10.1136/jmg.2004.024505

Kim, J. W., Zhang, H., Seymen, F., Koruyucu, M., Hu, Y., Kang, J., Kim, Y. J., Ikeda, A., Kasimoglu, Y., Bayram, M., Zhang, C., Kawasaki, K., Bartlett, J. D., Saunders, T. L., Simmer, J. P., and Hu, J. C. (2019). Mutations in RELT cause autosomal recessive amelogenesis imperfecta. *Clinical Genetics, 95*(3), 375-383. doi:10.1111/cge.13487

Kim, Y. J., Lee, Y., Chae, W., and Kim, J. W. (2023). Recessive COL17A1 Mutations and a Dominant LAMB3 Mutation Cause Hypoplastic Amelogenesis Imperfecta. *J Pers Med, 13*(10). doi:10.3390/jpm13101494

Lagerström, M., Dahl, N., Nakahori, Y., Nakagome, Y., Bäckman, B., Landegren, U., and Pettersson, U. (1991). A deletion in the amelogenin gene (AMG) causes X-linked amelogenesis imperfecta (AIH1). *Genomics, 10*(4), 971-975. doi:10.1016/0888-7543(91)90187-j

Lee, K. E., Ko, J., Le, C. G., Shin, T. J., Hyun, H. K., Lee, S. H., and Kim, J. W. (2015). Novel LAMB3 mutations cause non-syndromic amelogenesis imperfecta with variable expressivity. *Clinical Genetics, 87*(1), 90-92. doi:10.1111/cge.12340

Mátyás, G., Arnold, E., Carrel, T., Baumgartner, D., Boileau, C., Berger, W., and Steinmann, B. (2006). Identification and in silico analyses of novel TGFBR1 and TGFBR2 mutations in Marfan syndrome-related disorders. *Human Mutation, 27*(8), 760-769. doi:10.1002/humu.20353

McGrath, J. A., Gatalica, B., Li, K., Dunnill, M. G., McMillan, J. R., Christiano, A. M., Eady, R. A., and Uitto, J. (1996). Compound heterozygosity for a dominant glycine substitution and a recessive internal duplication mutation in the type XVII collagen gene results in junctional epidermolysis bullosa and abnormal dentition. *American Journal of Pathology, 148*(6), 1787-1796.

Nawaz, H., Parveen, A., Khan, S. A., Zalan, A. K., Khan, M. A., Muhammad, N., Hassib, N. F., Mostafa, M. I., Elhossini, R. M., Roshdy, N. N., Ullah, A., Arif, A., Khan, S., Ammerpohl, O., and Wasif, N. (2024). Brachyolmia, dental anomalies and short stature (DASS): Phenotype and genotype analyses of Egyptian and Pakistani patients. *Heliyon, 10*(1), e23688. doi:10.1016/j.heliyon.2023.e23688

O'Sullivan, J., Bitu, C. C., Daly, S. B., Urquhart, J. E., Barron, M. J., Bhaskar, S. S., Martelli-Júnior, H., dos Santos Neto, P. E., Mansilla, M. A., Murray, J. C., Coletta, R. D., Black, G. C., and Dixon, M. J. (2011). Whole-Exome sequencing identifies FAM20A mutations as a cause of amelogenesis imperfecta and gingival hyperplasia syndrome. *American Journal of Human Genetics, 88*(5), 616-620. doi:10.1016/j.ajhg.2011.04.005

Parry, D. A., Brookes, S. J., Logan, C. V., Poulter, J. A., El-Sayed, W., Al-Bahlani, S., Al Harasi, S., Sayed, J., Raïf el, M., Shore, R. C., Dashash, M., Barron, M., Morgan, J. E., Carr, I. M., Taylor, G. R., Johnson, C. A., Aldred, M. J., Dixon, M. J., Wright, J. T., Kirkham, J., Inglehearn, C. F., and Mighell, A. J. (2012). Mutations in C4orf26, encoding a peptide with in vitro hydroxyapatite crystal nucleation and growth activity, cause amelogenesis imperfecta. *American Journal of Human Genetics, 91*(3), 565-571. doi:10.1016/j.ajhg.2012.07.020

Parry, D. A., Poulter, J. A., Logan, C. V., Brookes, S. J., Jafri, H., Ferguson, C. H., Anwari, B. M., Rashid, Y., Zhao, H., Johnson, C. A., Inglehearn, C. F., and Mighell, A. J. (2013). Identification of mutations in SLC24A4, encoding a potassium-dependent sodium/calcium exchanger, as a cause of amelogenesis imperfecta. *American Journal of Human Genetics, 92*(2), 307-312. doi:10.1016/j.ajhg.2013.01.003

Parry, D. A., Smith, C. E., El-Sayed, W., Poulter, J. A., Shore, R. C., Logan, C. V., Mogi, C., Sato, K., Okajima, F., Harada, A., Zhang, H., Koruyucu, M., Seymen, F., Hu, J. C., Simmer, J. P., Ahmed, M., Jafri, H., Johnson, C. A., Inglehearn, C. F., and Mighell, A. J. (2016). Mutations in the pH-Sensing G-protein-Coupled Receptor GPR68 Cause Amelogenesis Imperfecta. *American Journal of Human Genetics, 99*(4), 984-990. doi:10.1016/j.ajhg.2016.08.020

Poulter, J. A., Brookes, S. J., Shore, R. C., Smith, C. E., Abi Farraj, L., Kirkham, J., Inglehearn, C. F., and Mighell, A. J. (2014a). A missense mutation in ITGB6 causes pitted hypomineralized amelogenesis imperfecta. *Human Molecular Genetics, 23*(8), 2189-2197. doi:10.1093/hmg/ddt616

Poulter, J. A., El-Sayed, W., Shore, R. C., Kirkham, J., Inglehearn, C. F., and Mighell, A. J. (2014b). Whole-exome sequencing, without prior linkage, identifies a mutation in LAMB3 as a cause of dominant hypoplastic amelogenesis imperfecta. *European Journal of Human Genetics, 22*(1), 132-135. doi:10.1038/ejhg.2013.76

Poulter, J. A., Murillo, G., Brookes, S. J., Smith, C. E., Parry, D. A., Silva, S., Kirkham, J., Inglehearn, C. F., and Mighell, A. J. (2014c). Deletion of ameloblastin exon 6 is associated with amelogenesis imperfecta. *Human Molecular Genetics, 23*(20), 5317-5324. doi:10.1093/hmg/ddu247

Prasad, M. K., Geoffroy, V., Vicaire, S., Jost, B., Dumas, M., Le Gras, S., Switala, M., Gasse, B., Laugel-Haushalter, V., Paschaki, M., Leheup, B., Droz, D., Dalstein, A., Loing, A., Grollemund, B., Muller-Bolla, M., Lopez-Cazaux, S., Minoux, M., Jung, S., Obry, F., Vogt, V., Davideau, J. L., Davit-Beal, T., Kaiser, A. S., Moog, U., Richard, B., Morrier, J. J., Duprez, J. P., Odent, S., Bailleul-Forestier, I., Rousset, M. M., Merametdijan, L., Toutain, A., Joseph, C., Giuliano, F., Dahlet, J. C., Courval, A., El Alloussi, M., Laouina, S., Soskin, S., Guffon, N., Dieux, A., Doray, B., Feierabend, S., Ginglinger, E., Fournier, B., de la Dure Molla, M., Alembik, Y., Tardieu, C., Clauss, F., Berdal, A., Stoetzel, C., Manière, M. C., Dollfus, H., and Bloch-Zupan, A. (2016). A targeted next-generation sequencing assay for the molecular diagnosis of genetic disorders with orodental involvement. *Journal of Medical Genetics, 53*(2), 98-110. doi:10.1136/jmedgenet-2015-103302

Rajpar, M. H., Harley, K., Laing, C., Davies, R. M., and Dixon, M. J. (2001). Mutation of the gene encoding the enamel-specific protein, enamelin, causes autosomal-dominant amelogenesis imperfecta. *Human Molecular Genetics, 10*(16), 1673-1677. doi:10.1093/hmg/10.16.1673

Seymen, F., Kim, Y. J., Lee, Y. J., Kang, J., Kim, T. H., Choi, H., Koruyucu, M., Kasimoglu, Y., Tuna, E. B., Gencay, K., Shin, T. J., Hyun, H. K., Kim, Y. J., Lee, S. H., Lee, Z. H., Zhang, H., Hu, J. C., Simmer, J. P., Cho, E. S., and Kim, J. W. (2016). Recessive Mutations in ACPT, Encoding Testicular Acid Phosphatase, Cause Hypoplastic Amelogenesis Imperfecta. *American Journal of Human Genetics, 99*(5), 1199-1205. doi:10.1016/j.ajhg.2016.09.018

Smith, C. E., Murillo, G., Brookes, S. J., Poulter, J. A., Silva, S., Kirkham, J., Inglehearn, C. F., and Mighell, A. J. (2016). Deletion of amelotin exons 3-6 is associated with amelogenesis imperfecta. *Human Molecular Genetics, 25*(16), 3578-3587. doi:10.1093/hmg/ddw203

Smith, C. E. L., Whitehouse, L. L. E., Poulter, J. A., Wilkinson Hewitt, L., Nadat, F., Jackson, B. R., Manfield, I. W., Edwards, T. A., Rodd, H. D., Inglehearn, C. F., and Mighell, A. J. (2020). A missense variant in specificity protein 6 (SP6) is associated with amelogenesis imperfecta. *Human Molecular Genetics, 29*(9), 1417-1425. doi:10.1093/hmg/ddaa041

Wang, S. K., Aref, P., Hu, Y., Milkovich, R. N., Simmer, J. P., El-Khateeb, M., Daggag, H., Baqain, Z. H., and Hu, J. C. (2013). FAM20A mutations can cause enamel-renal syndrome (ERS). *PLoS Genetics, 9*(2), e1003302. doi:10.1371/journal.pgen.1003302

Wang, S. K., Choi, M., Richardson, A. S., Reid, B. M., Lin, B. P., Wang, S. J., Kim, J. W., Simmer, J. P., and Hu, J. C. (2014). ITGB6 loss-of-function mutations cause autosomal recessive amelogenesis imperfecta. *Human Molecular Genetics, 23*(8), 2157-2163. doi:10.1093/hmg/ddt611

Yamaguti, P. M., Neves, F. A., Hotton, D., Bardet, C., de La Dure-Molla, M., Castro, L. C., Scher, M. D., Barbosa, M. E., Ditsch, C., Fricain, J. C., de La Faille, R., Figueres, M. L., Vargas-Poussou, R., Houillier, P., Chaussain, C., Babajko, S., Berdal, A., and Acevedo, A. C. (2017). Amelogenesis imperfecta in familial hypomagnesaemia and hypercalciuria with nephrocalcinosis caused by CLDN19 gene mutations. *Journal of Medical Genetics, 54*(1), 26-37. doi:10.1136/jmedgenet-2016-103956
