# Supplementary material for: Truncated Variants in FAM20A and WDR72 Genes Underlie Autosomal Recessive Amelogenesis Imperfecta in Four Pakistani Families
Source: Biochem Genet. 2025 Mar 19;64(1):1311–23. doi: 10.1007/s10528-025-11087-2 (PMC12882964; doi:10.1007/s10528-025-11087-2)
Supplement: Supplementary file 2 — Supplementary file2 (DOCX 19 KB)—Supplementary Table 2. Phenotypic characteristics of the affected individuals in families A, B, C, and D [file 10528_2025_11087_MOESM2_ESM.docx]

**Supplementary table 2.** Phenotypic characteristics of the affected individuals in families A, B, C and D

| **Family** | **A** | | | | **B** | | | | **C** | | **D** | |
| --- | --- | --- | --- | --- | --- | --- | --- | --- | --- | --- | --- | --- |
| **Probands** | IV-3 | IV-5 | IV-7 | IV-9 | IV-2 | IV-4 | IV-5 | IV-6 | V-1 | V-2 | IV-2 | IV-3 |
| **Sex** | F | F | F | F | M | M | M | M | F | F | M | M |
| **Age** | 18 | 16 | 6 | 5 | 28 | 23 | 18 | 15 | 6 | 4 | 14 | 13 |
| **Disease onset** | By birth | By birth | By birth | By birth | By birth | By birth | By birth | By birth | By birth | By birth | By birth | By birth |
| **Absent or thin hypoplastic**  **enamel** | + | + | + | + | + | + | + | + | + | + | - | - |
| **Abnormal tooth cusps** | + | + | + | + | + | + | + | + | + | + | - | - |
| **Gingival fibromatosis** | - | - | - | - | + | + | + | + | - | - | - | + |
| **Spaced teeth or relative microdontia** | + | + | + | + | + | + | + | + | + | + | - | - |
| **Delayed teeth eruptions** | + | + | + | + | + | + | + | + | + | + | + | + |
| **Root dilaceration of impacted enamel/teeth** | + | + | + | + | + | + | + | + | + | + | + | + |
| **Stained teeth** | + | + | + | + | + | + | + | + | + | + | + | + |
| **Enamel density** | + | + | + | + | + | + | + | + | + | + | - | - |
| **Hypodontia** | + | + | + | + | + | + | + | + | + | + | - | - |

‘+’ shows presence/abnormality, ‘-’ shows absence/normality, M: male, F: female
