# Supplementary material for: Truncated Variants in FAM20A and WDR72 Genes Underlie Autosomal Recessive Amelogenesis Imperfecta in Four Pakistani Families
Source: Biochem Genet. 2025 Mar 19;64(1):1311–23. doi: 10.1007/s10528-025-11087-2 (PMC12882964; doi:10.1007/s10528-025-11087-2)
Supplement: Supplementary file 3 — Supplementary file3 (DOCX 19 KB)—Supplementary Table 3. In-house AI gene panel used to detect the causative disease variants in AI patient during exome sequencing [file 10528_2025_11087_MOESM3_ESM.docx]

**Supplementary table 3.** In-house AI gene panel used to detect the causative disease variants in AI patient during exome sequencing

| **S. No.** | **Gene** | **OMIM** | **Genomic Location (GRCh38/hg38)** | **Cytogenic location** |
| --- | --- | --- | --- | --- |
| 1. | *AMELX* | 300391 | ChrX:11,293,413-11,309,588 | Xp22.2 |
| 2. | *ENAM* | 606585 | Chr4:71,494,461-71,512,541 | 4q13.3 |
| 3. | *KLK4* | 603767 | Chr19:51,409,607-51,413,994 | 19q13.41 |
| 4. | *DLX3* | 600525 | Chr17:49,990,005-49,995,224 | 17q21.33 |
| 5. | *MMP20* | 604629 | Chr11:102,447,563-102,496,063 | 11q22.2 |
| 6. | *FAM83H* | 611927 | Chr8:143,723,933-143,738,234 | 8q24.3 |
| 7. | *WDR72* | 613214 | Chr15:53,805,938-54,055,075 | 15q21.3 |
| 8. | *FAM20A* | 611062 | Chr17:68,535,113-68,601,367 | 17q24.2 |
| 9. | *ODAPH/C4orf26* | 614829 | Chr 4:75,556,048-75,565,893 | 4q21.1 |
| 10. | *SLC24A4* | 609840 | Chr14:92,322,581-92,501,481 | 14q32.12 |
| 11. | *LAMB3* | 150310 | Chr1:209,614,870-209,652,425 | 1q32.2 |
| 12. | *ITGB6* | 147558 | Chr2:160,956,182-161,056,783 | 2q24.2 |
| 13. | *AMBN* | 601259 | Chr4:70,592,256-70,607,288 | 4q13.3 |
| 14. | *ACPT* | 606362 | Chr19:50,790,415-50,795,219 | 19q13.33 |
| 15. | *GPR68* | 601404 | Chr14:91,232,532-91,270,790 | 14q32.11 |
| 16. | *AMTN* | 610912 | Chr4:70,518,569-70,532,743 | 4q13.3 |
| 17. | *RELT* | 611211 | Chr11:73,376,399-73,397,474 | 11q13.4 |
| 18. | *SP6* | 608613 | Chr17:47,844,908-47,876,311 | 17q21.32 |
