# Supplementary material for: Truncated Variants in FAM20A and WDR72 Genes Underlie Autosomal Recessive Amelogenesis Imperfecta in Four Pakistani Families
Source: Biochem Genet. 2025 Mar 19;64(1):1311–23. doi: 10.1007/s10528-025-11087-2 (PMC12882964; doi:10.1007/s10528-025-11087-2)
Supplement: Supplementary file 4 — Supplementary file4 (DOCX 14 KB)—Supplementary Table 4. Pathogenicity prediction of FAM20A and WDR72 variants by using different tools [file 10528_2025_11087_MOESM4_ESM.docx]

**Supplementary table 4.** Pathogenicity prediction of *FAM20A* and *WDR72* variants by using different tools

| S. No. | Gene | Variant | MutationTaster | VarSome | CADD | ACMG 2015 classification |
| --- | --- | --- | --- | --- | --- | --- |
| 1. | *FAM20A* | c.188dup; p. (Asp63Glufs*17) | Disease causing  Prob. Score: 1.00 | Pathogenic | Damaging  Score: 37 | Pathogenic  (PVS1, PM2, PM3, PP1, PP4, PP5) |
| 2. | *WDR72* | c.2686C>T; p. (Arg896*) | Disease causing  Prob. Score: 0.9999 | Pathogenic | NA | Pathogenic  (PVS1, PM2, PM3, PP1, PP4, PP5) |
